# Evaluation of Antibody Response to SARS-CoV-2 mRNA-1273 Vaccination in Patients With Cancer in Florida

**DOI:** 10.1001/jamaoncol.2022.0001

**Published:** 2022-03-10

**Authors:** Anna R. Giuliano, Jeffrey E. Lancet, Shari Pilon-Thomas, Ning Dong, Akriti G. Jain, Elaine Tan, Somedeb Ball, Shelley S. Tworoger, Erin M. Siegel, Junmin Whiting, Qianxing Mo, Christopher L. Cubitt, Christopher W. Dukes, Jonathan A. Hensel, Robert J. Keenan, Patrick Hwu

**Affiliations:** 1 Moffitt Cancer Center, Tampa, Florida

## Abstract

**Importance:**

Patients with cancer experience high rates of morbidity and mortality after SARS-CoV-2 infection. Immune response to mRNA-1273 vaccination across multiple cancer types and treatments remains to be established.

**Objective:**

To quantitate antibody responses after mRNA-1273 vaccination among patients with solid tumors and hematologic cancer and to assess clinical and treatment factors associated with vaccine response.

**Design, Setting, and Participants:**

This cohort study included patients with cancer who were aged 18 years or older, spoke English or Spanish, had received their first mRNA-1273 dose between January 12 and 25, 2021, and agreed to blood tests before and after vaccination.

**Exposures:**

Receipt of 1 and 2 mRNA-1273 SARS-CoV-2 vaccine doses.

**Main Outcomes and Measures:**

Seroconversion after each vaccine dose and IgG levels against SARS-CoV-2 spike protein obtained immediately before the first and second vaccine doses and 57 days (plus or minus 14 days) after the first vaccine dose. Cancer diagnoses and treatments were ascertained by medical record review. Serostatus was assessed via enzyme-linked immunosorbent assay. Paired *t* tests were applied to examine days 1, 29, and 57 SARS-CoV-2 antibody levels. Binding antibody IgG geometric mean titers were calculated based on log_10_-transformed values.

**Results:**

The 515 participants were a mean (SD) age of 64.5 (11.4) years; 262 (50.9%) were women; and 32 (6.2%) were Hispanic individuals and 479 (93.0%) White individuals; race and ethnicity data on 4 (0.7%) participants were missing. Seropositivity after vaccine dose 2 was 90.3% (465; 95% CI, 87.4%-92.7%) among patients with cancer, was significantly lower among patients with hematologic cancer (84.7% [255]; 95% CI, 80.1%-88.6%) vs solid tumors (98.1% [210]; 95% CI, 95.3%-99.5%), and was lowest among patients with lymphoid cancer (70.0% [77]; 95% CI, 60.5%-78.4%). Patients receiving a vaccination within 6 months after anti-CD20 monoclonal antibody treatment had a significantly lower seroconversion (6.3% [1]; 95% CI, 0.2%-30.2%) compared with those treated 6 to 24 months earlier (53.3% [8]; 95% CI, 26.6%-78.7%) or those who never received anti-CD20 treatment (94.2% [456]; 95% CI, 91.7%-96.1%). Low antibody levels after vaccination were observed among patients treated with anti-CD20 within 6 months before vaccination (GM, 15.5 AU/mL; 95% CI, 9.8-24.5 AU/mL), patients treated with small molecules (GM, 646.7 AU/mL; 95% CI, 441.9-946.5 AU/mL), and patients with low lymphocyte (GM, 547.4 AU/mL; 95% CI, 375.5-797.7 AU/mL) and IgG (GM, 494.7 AU/mL; 95% CI, 304.9-802.7 AU/mL) levels.

**Conclusions and Relevance:**

This cohort study found that the mRNA-1273 SARS-CoV-2 vaccine induced variable antibody responses that differed by cancer diagnosis and treatment received. These findings suggest that patients with hematologic cancer and those who are receiving immunosuppressive treatments may need additional vaccination doses.

## Key Points

**Question:**

Does the immune response to the mRNA-1273 vaccine differ among patients with solid tumors and hematologic cancer?

**Findings:**

In this cohort study of 515 patients with cancer, seropositivity after the first and second vaccine doses was 71% and 90%, respectively. Antibody levels after vaccination were substantially higher among patients who were seropositive before vaccination.

**Meaning:**

Results of this study suggest that the mRNA-1273 vaccine induced a highly variable seroconversion percentage among patients with cancer; these patients may benefit from additional vaccine doses.

## Teaser

This cohort study investigates the immune response of patients with cancer to the first and second doses of the COVID-19 mRNA-1273 vaccination.

## Introduction

Patients with cancer have many risk factors for poor SARS-CoV-2 infection outcomes,^1^ underscoring an urgency for vaccination. Some reports have indicated suboptimal responses to vaccination among patients with cancer, although sample sizes were small, limiting comparisons across disease and treatment characteristics.^2,3^ The humoral response kinetics to the mRNA-1273 vaccination among patients with cancer has not been fully evaluated.

We conducted an observational study with the primary and secondary aims of quantitating antibody responses before and after SARS-CoV-2 mRNA-1273 vaccination among patients diagnosed with solid tumors and hematologic cancer and to assess clinical and treatment factors associated with antibody levels after vaccination. We also assessed whether antibody status before vaccination was associated with antibody levels achieved after 2 vaccine doses.

## Methods

All of the patients in this cohort study had cancer and were sequentially enrolled from those presenting for mRNA-1273 vaccination at Moffitt Cancer Center between January 12 and 25, 2021. Patients provided one 10-mL tiger-top blood sample before the first and second vaccine doses (days 1 and 29) and on day 57 (plus or minus 14 days). Patients met study eligibility requirements if they provided written informed consent to Total Cancer Care; were aged 18 years or older; spoke English or Spanish; had received their first mRNA-1273 dose at the Moffitt Cancer Center between January 12 and 25, 2021; and agreed to blood tests before and after vaccination. Patients were excluded if they did not provide consent, declined the blood draws, or indicated they could not attend visits after vaccination. Blood collection occurred under an Advarra Institutional Review Board–approved Total Cancer Care protocol; specimen retrieval and analyses occurred under a separate protocol. This study followed the Strengthening the Reporting of Observational Studies in Epidemiology (STROBE) reporting guideline.

A total of 863 patients were approached, 690 were enrolled, and 175 were excluded, with a final sample size of 515 patients (eFigure in the Supplement). The patients self-reported race and ethnicity. Retrospective medical record review was used to ascertain cancer diagnoses and treatments.

Serostatus was assessed via enzyme-linked immunosorbent assay adapted from the Krammer protocol.^4,5^ Negative controls included serum pools from individuals witout cancer collected before 2015. Positive controls included convalescent serum from patients without cancer who tested positive for COVID-19. Antibody levels were quantitated using the Human SARS-CoV-2 Serology Standard provided by the Frederick National Laboratory for Cancer Research. eTable 1 in the Supplement includes details and quality control results.

For comparison, antibody levels obtained 14 to 60 days after mRNA-1273 vaccination from 18 adults without cancer aged 24 to 72 years, who participated in a separate community study performed by some of us, were quantitated.^6^

### Statistical Analysis

Patient characteristics were summarized using descriptive statistics (mean [SD] for continuous variables and proportions and frequencies for categorical measures). SARS-CoV-2 antibody positivity was compared across patient characteristics using the Fisher exact or χ^2^ test. The association of SARS-CoV-2 antibody levels and patient characteristics was examined using the Kruskal-Wallis test. Paired *t* tests were applied to examine SARS-CoV-2 antibody levels on days 1, 29, and 57. Binding antibody IgG geometric mean titers were calculated based on log_10_-transformed values. Observations with missing data were removed from the analysis. All analyses were performed using SAS, version 9.4 (SAS Institute, Inc) and R software, version 4.0.2 (R Foundation for Statistical Computing). Raw *P* values were corrected for multiple comparisons using the Bonferroni method, and adjusted 2-sided *P* < .05 was considered to be statistically significant.

## Results

Of 515 participants with a mean (SD) age of 64.5 (11.4) years, 262 (50.9%) were women, 253 (49.1%) were men, 32 (6.2%) were Hispanic individuals, and 479 (93.0%) were White individuals (race and ethnicity data on 4 [0.7%] participants were missing). There were 301 (58.4%) patients with hematologic cancer and 214 (41.6%) with solid tumors. Seventeen (3.3%) patients were SARS-CoV-2 seropositive before vaccination (Table 1).

**Table 1.** Seropositivity Percentages Before Vaccination and After Receipt of 1 and 2 Vaccine Doses Stratified by Patient and Cancer Treatment Characteristics

Characteristic	No. of patients	No. seropositive (%) [95% CI]^a^				
		Day 1	After dose 1	Adjusted *P* value^b^	After dose 2	Adjusted *P* value^b^
Overall	515	17 (3.3) [1.9-5.2]	367 (71.3) [67.1-75.1]		465 (90.3) [87.4-92.7]	
Age group, y						
<66	253	9 (3.6) [1.6-6.6]	194 (76.7) [71-81.7]	.20	231 (91.3) [87.1-94.5]	.99
≥66	262	8 (3.1) [1.3-5.9]	173 (66.0) [59.9-71.7]		234 (89.3) [84.9-92.8]	
Cancer diagnosis^c^						
Hematologic cancer^d^	301	11 (3.7) [1.8-6.4]	181 (60.1) [54.4-65.7]	<.001	255 (84.7) [80.1-88.6]	<.001
Myeloid^e^	93	2 (2.2) [0.3-7.6]	61 (65.6) [55-75.1]	.31	86 (92.5) [85.1-96.9]	<.001
Lymphoid	110	3 (2.7) [0.6-7.8]	54 (49.1) [39.4-58.8]		77 (70.0) [60.5-78.4]	
Plasma cell	98	6 (6.1) [2.3-12.9]	66 (67.3) [57.1-76.5]		92 (93.9) [87.1-97.7]	
Solid tumors	214	6 (2.8) [1.0- 6.0]	186 (86.9) [81.6-91.1]	<.001	210 (98.1) [95.3-99.5]	<.001
Disease status^f^						
Previously untreated	41	0	34 (82.9) [67.9-92.8]	.25	41 (100) [91.4-100]	.66
Remission	289	12 (4.2) [2.2-7.1]	215 (74.4) [69.0-79.3]		262 (90.7) [86.7-93.8]	
Relapse, refractory, or stable disease	184	5 (2.7) [0.9-6.2]	117 (63.6) [56.2-70.5]		161 (87.5) [81.8-91.9]	
Lymphocyte count^g^						
>1 × 10^9^/L	311	12 (3.9) [1.7-6.0]	236 (75.9) [71.1-80.6]	<.001	288 (92.6) [89.7-95.5]	.01
≤1 × 10^9^/L	141	4 (2.8) [0.1-5.6]	75 (53.2) [45.0-61.4]		115 (81.6) [75.2-88.0]	
Immunoglobulin levels with plasma cell disorder and CLL (n = 121)						
IgG level^g^						
< 700 mg/dL	51	1 (2.0) [0-10.4]	24 (47.1) [32.9-61.5]	.001	47 (92.2) [81.1-97.8]	.99
≥700 mg/dL	49	5 (10.2) [3.4-22.2]	42 (85.7) [72.8-94.1]		46 (93.9) [83.1-98.7]	
IgA level^g^						
<70 mg/dL	53	3 (5.7) [1.2-15.7]	27 (50.9) [36.8-64.9]	.01	48 (90.6) [79.3-96.9]	.99
≥70 mg/dL	46	3 (6.5) [1.4-17.9]	39 (84.8) [71.1-93.7]		44 (95.7) [85.2-99.5]	
IgM level^g^						
< 40 mg/dL	75	5 (6.7) [2.2-14.9]	44 (58.7) [46.7-69.9]	.07	68 (90.7) [81.7-96.2]	.99
≥ 40 mg/dL	24	1 (4.2) [0.1-21.1]	22 (91.7) [73.0-99.0]		24 (100) [85.8-100]	
Received anticancer therapy within 3 mo^h^						
No	275	9 (3.3) [1.5-6.1]	211 (76.7) [71.3-81.6]	.09	255 (92.7) [89.0-95.5]	.99
Yes	240	8 (3.3) [1.4-6.5]	156 (65.0) [58.6-71.0]		210 (87.5) [82.6-91.4]	
Received small molecules within 3 mo^i^						
No	396	11 (2.8) [1.4-4.9]	290 (73.2) [68.6-77.5]	.99	361 (91.2) [87.9-93.8]	.99
Yes	119	6 (5.0) [1.9-10.7]	77 (64.7) [55.4-73.2]		104 (87.4) [80.1-92.8]	
Received anti-CD20 monoclonal antibodies						
Within 6 mo before dose	16	0	0	<.001	1 (6.3) [0.2-30.2]	<.001
6-24 mo Before dose	15	0	3 (20.0) [4.3-48.1]		8 (53.3) [26.6-78.7]	
Not treated with anti-CD20	484	17 (3.5) [2.1-5.6]	364 (75.2) [71.1-79.0]		456 (94.2) [91.7-96.1]	
Received anti-CD38 antibodies						
Within 6 mo before dose	47	1 (2.1) [0.1-11.3]	27 (57.4) [42.2-71.7]	.51	41 (87.2) [74.3-95.2]	.99
6-24 mo Before dose	12	0	6 (50.0) [21.1-78.9]		12 (100) [73.5-100]	
Not treated with anti-CD38	456	16 (3.5) [2.0-5.6]	334 (73.2) [68.9-77.3]		412 (90.4) [87.3-92.9]	
Received cellular therapy						
Allo-HSCT any time before vaccination	63	1 (1.6) [0-8.5]	36 (57.1) [44-69.5]	NA	58 (92.1) [82.4-97.4]	NA
Auto-HSCT within the past 12 mo	19	2 (10.5) [1.3-33.1]	15 (78.9) [54.4-93.9]		19 (100) [82.4-100]	
Line of systemic therapy to date						
0	64	3 (4.7) [0.4-10.8]	57 (89.1) [78.8-95.5]	<.001	64 (100) [94.4-100]	.004
1	229	11 (4.8) [2.1-7.9]	174 (76.0) [69.9-81.4]		212 (92.6) [88.4-95.6]	
≥2	222	5 (2.3) [0.7-5.2]	136 (61.3) [54.5-67.7]		189 (85.1) [79.8-89.5]	

Abbreviations: Allo-HSCT, allogeneic hematopoietic stem cell transplant; auto-HSCT, autologous hematopoietic stem cell transplant; CLL, chronic lymphocytic leukemia; NA, not applicable.

SI conversion: to convert immunoglobulin level to g/L, multiply by 0.01.

^a^ Blood draws were conducted on day 1, before the first vaccine dose; day 29, after the first dose and before the second dose; and day 57, which was 4 weeks (plus or minus 14 days) after the second dose.

^b^ *P* values were calculated comparing patients receiving a specific therapy with patients not receiving the therapy at a specific study time point by Fisher exact test or χ^2^ test and then adjusting for multiple comparisons using the Bonferroni method.

^c^ Fifty-nine patients had multiple cancers and were categorized according to the most active or severe cancer in the study investigators’ opinion.

^d^ *P* values were calculated comparing hematologic cancer with solid tumors.

^e^ *P* values were calculated comparing myeloid, lymphoid, and plasma cell neoplasms.

^f^ Disease status was missing for 1 patient.

^g^ All laboratory values were measured within 3 months before the first dose of vaccine. Sixty-three patients (12.2%) were missing a lymphocyte count. Among patients with plasma cell disorder or CLL, 21 (17.4%) were missing an IgG level, 22 (18.2%) were missing an IgA level, and 22 (18.2%) were missing an IgM level.

^h^ Antiandrogen and antiestrogen hormonal therapies were not considered anticancer therapy for the purpose of this study.

^i^ Small molecules include tyrosine kinase inhibitors, proteasome inhibitors, lenalidomide, pomalidomide, and venetoclax.

Overall, 71.3% (367; 95% CI, 67.1%-75.1%) and 90.3% (465; 95% CI, 87.4%-92.7%) of patients seroconverted after 1 and 2 vaccine doses, respectively (Table 1). Seroconversion after the second vaccine dose was lower among patients with hematologic cancer vs solid tumors (84.7% [255]; 95% CI, 80.1%-88.6% vs 98.1% [210]; 95% CI, 95.3%-99.5%). Within the hematologic cancer category, patients with lymphoid cancer had a low seroconversion percentage (70.0% [77]; 95% CI, 60.5%-78.4%), particularly among patients with chronic lymphocytic leukemia and B-cell non-Hodgkin lymphoma (65.2% [15 of 23] and 58.2% [32 of 55], respectively; eTable 2 in the Supplement). The response was lowest among patients with chronic lymphocytic leukemia and B-cell non-Hodgkin lymphoma receiving treatment (30.4% [7 of 23]) vs not receiving treatment (72.7% [40 of 55]). Patients treated with anti-CD20 monoclonal antibodies within 6 months before vaccination had a significantly lower seroconversion percentage (6.3% [1]; 95% CI, 0.2%-30.2%) vs patients who received treatment 6 to 24 months before vaccination (53.3% [8]; 95% CI, 26.6%-78.7%) and those not receiving this treatment (94.2% [456]; 95% CI, 91.7%-96.1%). After 2 vaccine doses, a low seroconversion percentage was also observed among patients treated with Bruton tyrosine kinase (BTK) inhibitors (33.3% [2 of 6]; 95% CI, 4.3%-77.7%), PI3K inhibitors (0%), or venetoclax (50.0% [3 of 6]; 95% CI, 11.8%-88.2%), and patients who underwent CD19 chimeric antigen receptor T-cell (CAR-T) therapy (12.5% [1 of 8]; 95% CI, 0.3%-52.7%; eTable 2 in the Supplement). In contrast, 100% of patients with autologous transplant treated within 12 months and those treated with B-cell maturation antigen CAR-T seroconverted after 2 doses (Table 1; and eTable 2 in the Supplement). Seroconversion percentages among patients with allogeneic hematopoietic stem cell transplant were high.

After vaccination, significantly higher antibody levels were observed among adults without cancer who participated in a separate community study^6^ (geometric mean [GM], 7303.7 arbitrary units (AU)/mL; 95% CI, 3906.8-13 654.2 AU/mL) vs patients with solid tumors (GM, 1754.6 AU/mL; 95% CI, 1502.7-2048.8 AU/mL) or hematologic cancer (GM, 745.6 AU/mL; 95% CI, 579.0-960.2 AU/mL), and among patients with cancer who were seropositive on day 1 (GM, 6821.0 AU/mL; 95% CI, 3530.1-13 179.4 AU/mL) vs seronegative (GM, 998.6 AU/mL; 95% CI, 845.4-1179.6 AU/mL) (Figure). Low antibody levels after vaccination were observed among patients with low lymphocyte (GM, 547.4 AU/mL; 95% CI, 375.7-797.7 AU/mL) and low IgG levels (GM, 494.7 AU/mL; 95% CI, 304.9-802.7 AU/mL) and among patients treated with anti-CD20 monoclonal antibodies within 6 months before vaccination (GM, 15.5 AU/mL; 95% CI, 9.8-24.5 AU/mL) or treated with small molecules, including tyrosine kinase inhibitors, proteasome inhibitors, lenalidomide, pomalidomide, and ventoclax (GM, 646.7; 95% CI, 441.9-946.5 AU/mL) (Table 2). eTable 3 in the Supplement shows the large range of achieved antibody levels after vaccination stratified by cancer diagnosis and treatment type, with especially low levels observed among patients with chronic lymphocytic leukemia and those treated with BTK inhibitors and venetoclax.

**Figure.**
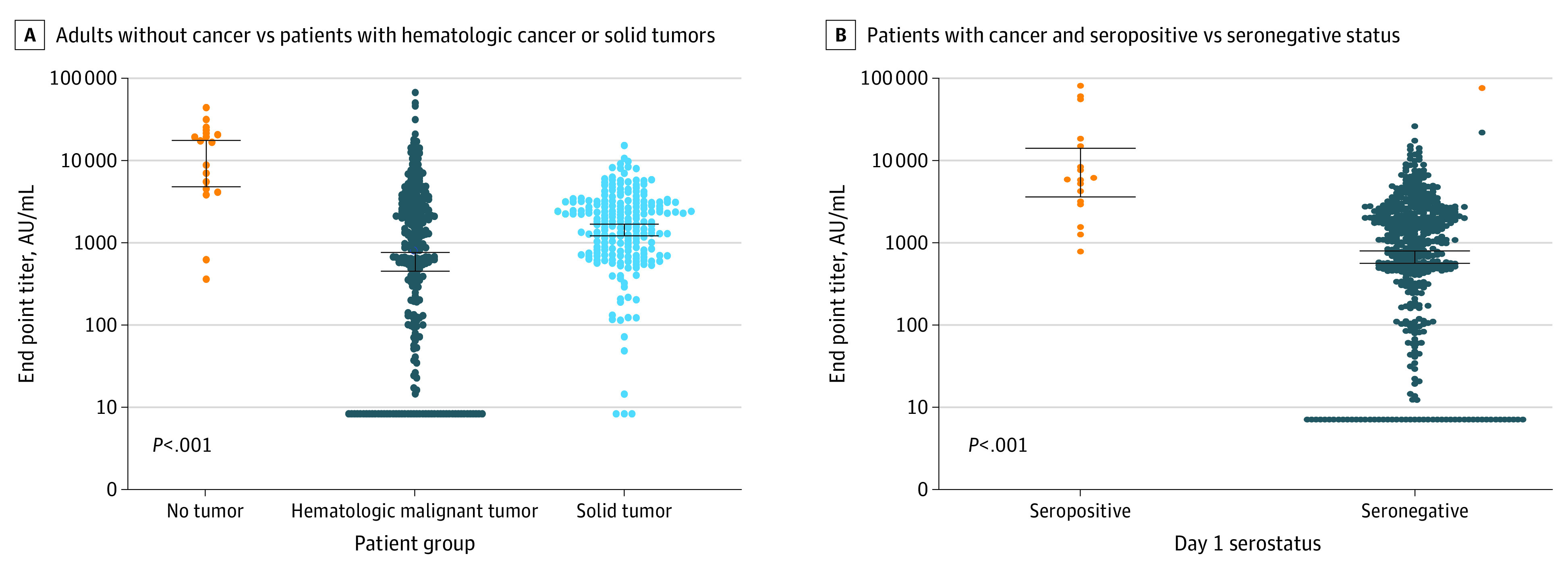
Antibody Titer Kinetics 28 Days After 2 Vaccine Doses Figure shows the antibody levels among 18 adults without cancer who participated in a separate community study^6^ compared with those of 301 patients with hematologic cancer and 214 patients with solid tumors (A) as well as the antibody levels among 17 patients with cancer and seropositive status vs 498 with cancer and seronegative status. AU indicates arbirary units.

**Table 2.** Antibody Geometric Mean (95% CI) Before Vaccination and After Receipt of 1 and 2 Vaccine Doses Stratified by Patient and Treatment Characteristics

Characteristic	Antibody geometric mean levels, AU/mL (95% CI)^a^				
	Day 1	After dose 1	*P* value^b^	After dose 2	*P* value^b^
Overall (n = 515)	13.7 (13.0-14.3)	98.2 (84.7-113.9)		1064.0 (902.3-1254.8)	
Age group, y					
<66	13.6 (12.8-14.5)	134.8 (109.2-166.4)	<.001	1334.6 (1061.7-1677.6)	.01
≥66	13.7 (12.8-14.7)	72.4 (59-88.7)		854.9 (675.4-1082.2)	
Cancer diagnosis^c^					
Hematologic cancer^d^	14.0 (13-15)	66.5 (54.4-81.3)	<.001	745.6 (579-960.2)	.009
Myeloid^e^	13.5 (11.9-15.2)	67.3 (47.2-95.8)	.99	1285.8 (858.6-1925.7)	.11
Lymphoid	13.4 (12.2-14.7)	59.5 (41.5-85.2)		396.3 (242.9-646.7)	
Plasma cell disorder	15.0 (12.8-17.7)	74.5 (53.2-104.2)		903.6 (624-1308.6)	
Solid tumors	13.3 (12.6-13.9)	170.1 (139.6-207.3)	<.001	1754.6 (1502.7-2048.8)	.009
Disease status^f^					
Previously untreated	12.5^g^	168.2 (102.9-275.1)	.01	2021.1 (1470.6-2777.7)	.001
Remission	13.8 (12.9-14.7)	111.3 (91.5-135.3)		1265.0 (1009.4-1585.4)	
Relapse/refractory/stable disease	13.8 (12.6-15)	71.0 (55.2-91.4)		701.0 (530.5-926.4)	
Lymphocyte count^h^					
>1000/uL	14.0 (13.0-15.0)	117.4 (96.9-142.3)	<.001	1321.5 (1084.1-1611)	.002
≤1000/uL	13.4 (12.4-14.5)	49.4 (37.6-64.7)		547.4 (375.7-797.7)	
Immunoglobulin levels with plasma cell disorder and CLL (n = 121)					
IgG level^h^					
< 700 mg/dL	13.4 (11.6-15.5)	35.0 (24.5-50.1)	<.001	494.7 (304.9-802.7)	.01
≥700 mg/dL	16.8 (12.6-22.4)	148.3 (89.9-244.6)		1496.1 (892.1-2508.9)	
IgA level^h^					
<70 mg/dL	15.3 (12.1-19.5)	37.8 (25.2-56.5)	<.001	591.7 (362.7-965.3)	.32
≥70 mg/dL	14.7 (11.8-18.1)	152.6 (94.6-246.2)		1346.2 (782.7-2315.3)	
IgM level^h^					
< 40 mg/dL	15.4 (12.6-18.9)	51.3 (35.3-74.4)	.002	669.6 (431.8-1038.5)	.25
≥40 mg/dL	13.8 (11.3-16.8)	211.2 (116.2-383.6)		1942.6 (1106.3-3411.3)	
Received anticancer therapy within 3 mo^i^					
No	13.6 (12.8-14.5)	116.9 (96.1-142.2)	.09	1450.4 (1182-1779.6)	.003
Yes	13.7 (12.8-14.7)	80.4 (64.3-100.7)		746.1 (575.6-967.1)	
Received small molecules within 3 mo^j^					
No	13.4 (12.8-14.0)	107.3 (90.7-126.9)	0.33	1235.8 (1032.5-1479.1)	.01
Yes	14.6 (12.8-16.7)	73.2 (53.4-100.2)		646.7 (441.9-946.5)	
Received anti-CD20 monoclonal antibodies					
Within 6 mo before dose	12.5^g^	12.5^g^	<.001	15.5 (9.8-24.5)	<.001
6-24 mo Before dose	12.5^g^	29.3 (12.1-71)		345.2 (60.1-1983.4)	
Not treated with anti-CD20	13.7 (13.1-14.4)	109.2 (93.9-126.9)		1267.1 (1088.7-1474.8)	
Received anti-CD38 antibodies					
Within 6 mo before dose	13.5 (11.6-15.7)	52.7 (33.2-83.4)	.12	369.7 (219.7-622.1)	<.001
6-24 mo Before dose	12.5^g^	44.4 (17.4-113.2)		903.6 (292.3-2793.5)	
Not treated with anti-CD38	13.7 (13.0-14.4)	107.0 (91.3-125.3)		1191.6 (1001.3-1418.2)	
Received cellular therapy					
Allo-HSCT any time before vaccination	13.6 (11.5-16.2)	47.7 (32.1-71)	NA	1290.0 (762.0-2183.6)	NA
Auto-HSCT within the past 12 mo	20.0 (10.1-39.5)	166.4 (60.7-456.1)		2452.6 (1080.1-5569.1)	
Line of systemic therapy to date					
0	13.3 (12.1-14.6)	204.5 (142.2-294.1)	<.001	2097.1 (1662.2-2645.9)	.05
1	14.3 (13-15.7)	116.2 (92.9-145.3)		1315.5 (1041.1-1662.2)	
≥2	13.1 (12.6-13.7)	66.9 (53.5-83.6)		703.0 (530.8-931)	

Abbreviations: Allo-HSCT, allogeneic hematopoietic stem cell transplant; auto-HSCT, autologous hematopoietic stem cell transplant; CLL, chronic lymphocytic leukemia; NA, not applicable.

SI conversion: to convert immunoglobulin levels to g/L, multiply by 0.01.

^a^ Blood draws were conducted on day 1, before the first vaccine dose; day 29, after the first dose and before the second dose; and day 57, which was 4 weeks (plus or minus 14 days) after the second dose.

^b^ *P* values were calculated comparing patients receiving a specific therapy with patients not receiving the therapy at a specific study time point by Kruskal-Wallis test and then adjusting for multiple comparisons using the Bonferroni method.

^c^ Fifty-nine patients had multiple cancers and were categorized according to the most active or severe cancer in the study investigators’ opinion.

^d^ *P* values were calculated comparing hematologic cancer with solid tumors.

^e^ *P* values were calculated comparing myeloid, lymphoid, and plasma cell neoplasms.

^f^ Disease status was missing for 1 patient.

^g^ The antibody levels were too low for some patients and could not be detected; we have assigned a value of 12.5 for those patients. The 95% CI cannot be calculated if they are the same values.

^h^ All laboratory values were measured within 3 months before the first dose of vaccine. Sixty-three (12.2%) of total patients were missing lymphocyte count. Among patients with plasma cell disorder or CLL, 21 (17%) were missing an IgG level, 22 (18.2%) were missing an IgA level, and 22 (18%) were missing an IgM level.

^i^ Antiandrogen and antiestrogen hormonal therapies were not considered anticancer therapy for the purpose of this study.

^j^ Small molecules include tyrosine kinase inhibitors, proteasome inhibitors, lenalidomide, pomalidomide, and venetoclax.

## Discussion

This large study of patients with cancer enabled direct comparisons across multiple cancer diagnoses and treatments in patients who received 2 mRNA-1273 vaccine doses. Chronic lymphocytic leukemia and B-cell non-Hodgkin lymphoma had the lowest seroconversion percentages and antibody levels, consistent with other studies.^7^ Noteworthy was the complete lack of an antibody response in patients treated with anti-CD20 monoclonal antibodies and low seroconversion percentages among those treated with BTK inhibitors, venetoclax, and CD19-CAR-T. After the second dose, antibody levels were higher among patients who were seropositive at baseline compared with those who were seronegative. After 2 vaccine doses, patients who had a seropositive status before vaccination achieved antibody levels similar to those of adults without cancer, suggesting that patients with cancer with initially poor immune responses may benefit from additional vaccine doses, including a third dose, as recently recommended.^8^

Hematologic cancer is characterized by B-cell defects associated with lower rates of antibody response to vaccines, even in the absence of anticancer therapy.^9,10^ Seroconversion percentages among patients with allogeneic hematopoietic stem cell transplant were high. Patients receiving CD19-CAR-T had low seroconversion percentages, though most received CAR-T more than 1 year before the study, were in remission, and had not received cancer treatment in the past year, suggesting prolonged immunosuppression after CD19-CAR-T therapy.

Antibody levels required to confer protection against SARS-CoV-2 are unknown. However, lower initial antibody levels are of concern because levels decline over time,^11^ higher levels are needed to neutralize variants of concern,^12,13,14^ and new variants continue to emerge. Patients who had seropositive status before the start of vaccination had antibody levels similar to levels after 1 vaccine dose and substantially higher levels after 2 doses than patients with initially seronegative status. It remains to be tested whether a third dose will overcome the poor vaccine response among patients who did not seroconvert or had very low antibody levels.^15^

### Limitations

This study has limitations. Despite having a large sample size of patients with cancer, we were limited in our sample of patients who received certain targeted therapies (eg, BTK inhibitors, venetoclax, CD19-CAR-T, and B-cell maturation antigen CAR-T).

## Conclusions

Findings of this cohort study suggest that patients with solid tumors or hematologic cancer have an immune response after COVID-19 vaccination, although with lower antibody levels than adults without cancer who participated in a separate community study.^6^ Patients with hematologic cancer and those who are receiving immunosuppressive treatments may need to be given priority for the third dose of vaccination, with careful consideration given to the timing of vaccination relative to the receipt of cancer treatment.^8^

## Notes

Supplement. **eFigure.** Study Flowchart. **eTable 1.** Mean Intra-Assay Coefficient of Variation for SARS-CoV-2 Spike IgG Titers (Positive Samples). **eTable 2.** Percent Seropositive Before Vaccination and After Receipt of 1 and 2 Vaccine Doses. **eTable 3.** Antibody Levels Before Vaccination and After Receipt of 1 and 2 Vaccine Doses.
