# Supplementary material for: Evaluation of Antibody Response to SARS-CoV-2 mRNA-1273 Vaccination in Patients With Cancer in Florida
Source: JAMA Oncol. 2022 Mar 10;8(5):748–54. doi: 10.1001/jamaoncol.2022.0001 (PMC8914884; doi:10.1001/jamaoncol.2022.0001)
Supplement: Supplement. — eFigure. Study Flowchart. eTable 1. Mean Intra-Assay Coefficient of Variation for SARS-CoV-2 Spike IgG Titers (Positive Samples). eTable 2. Percent Seropositive Before Vaccination and After Receipt of 1 and 2 Vaccine Doses. eTable 3. Antibody Levels Before Vaccination and After Receipt of 1 and 2 Vaccine Doses. [file jamaoncol-e220001-s001.pdf]

Supplementary Online Content

Giuliano AR, Lancet JE, Pilon-Thomas S, et al. Evaluation of antibody response to SARS-CoV-2 mRNA-1273 Vaccination in Patients With Cancer in Florida. *JAMA Oncol*. Published online March 10, 2022. doi:10.1001/jamaoncol.2022.0001

eFigure. Study Flowchart
eTable 1. Mean Intra-Assay Coefficient of Variation for SARS-CoV-2 Spike IgG Titers (Positive Samples)
eTable 2. Percent Seropositive Before Vaccination and After Receipt of 1 and 2 Vaccine Doses
eTable 3. Antibody Levels Before Vaccination and After Receipt of 1 and 2 Vaccine Doses

This supplementary material has been provided by the authors to give readers additional information about their work.

© 2022 Giuliano AR et al. *JAMA Oncology.*

eFigure. Study Flowchart

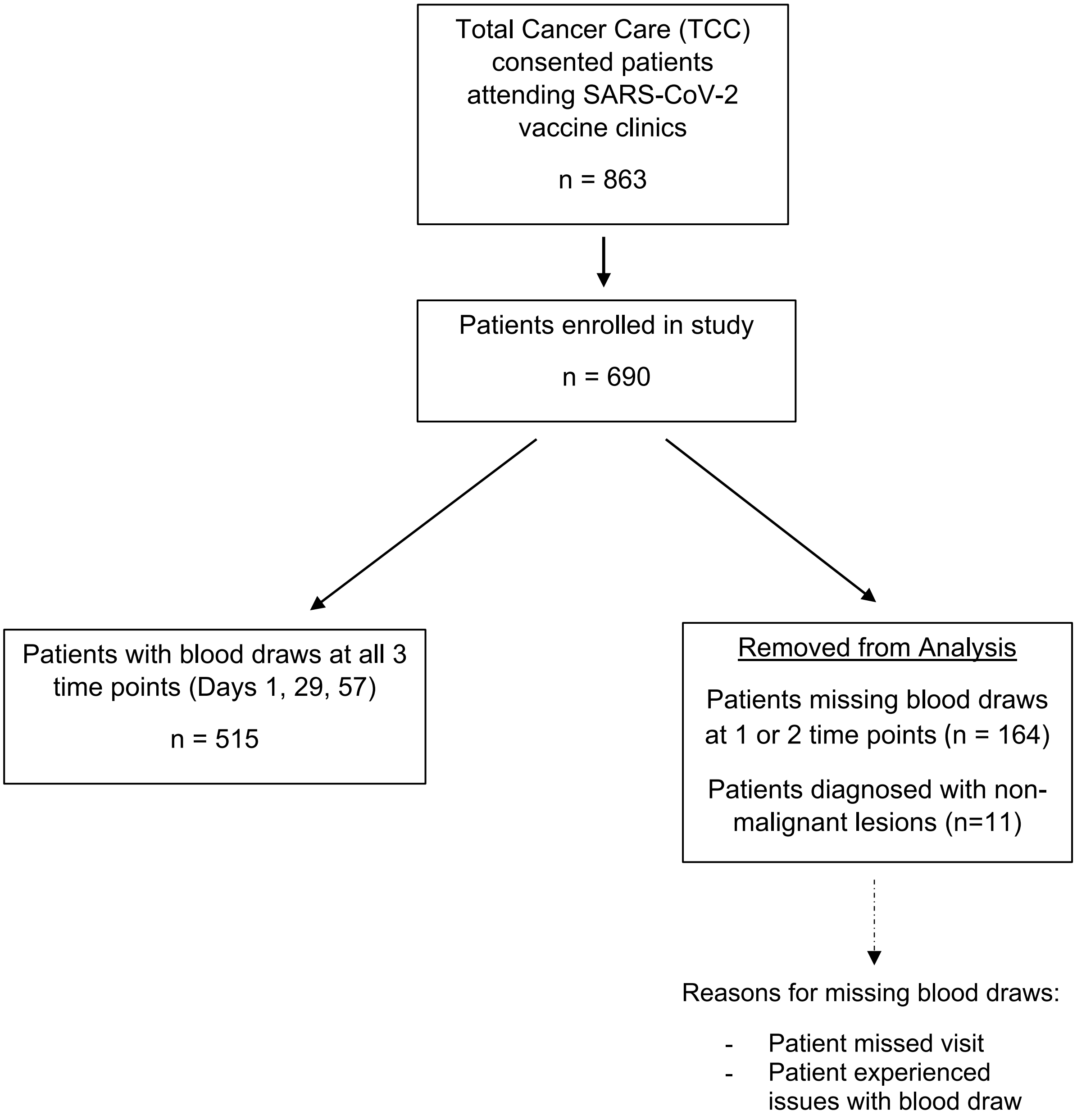

© 2022 Giuliano AR et al. *JAMA Oncology.*

eTable 1. Mean Intra-Assay Coefficient of Variation for SARS-CoV-2 Spike IgG Titers (Positive Samples)

Plate	Intra-Assay %CV	Plate	Intra-Assay %CV	Plate	Intra-Assay %CV	Plate	Intra-Assay %CV	Plate	Intra-Assay %CV
1	4.63	12	9.53	23	6.72	34	7.33	45	7.12
2	4.20	13	8.06	24	8.82	35	4.50	46	5.98
3	3.92	14	6.79	25	5.01	36	5.42	47	3.75
4	4.51	15	7.63	26	7.59	37	4.41	48	7.77
5	4.76	16	7.93	27	8.51	38	4.84	49	2.52
6	8.89	17	12.51	28	5.41	39	6.33	50	7.47
7	5.88	18	10.42	29	4.79	40	3.26	51	7.96
8	7.36	19	5.68	30	8.06	41	8.73	52	1.30
9	11.68	20	7.04	31	4.00	42	6.23	53	3.71
10	10.98	21	7.31	32	6.40	43	4.97	54	4.83
11	10.14	22	6.71	33	8.51	44	7.05	55	3.30

SARS-CoV-2 Antibody methods

Serum samples to be tested were aliquoted into a 96-well polypropylene source plate by adding 5µL of serum into 95µL of 1X PBS in the respective well. These plates were provided to the core facility and were tested within 24 hours of receipt. On the day of assays, another 96-well source plate was created that included negative and positive controls as well as a set of internal standards consisting of a 12-point 2-fold dilution series beginning at a 1:20 dilution of the standard. The negative control consisted of a pool of 41 serum samples confirmed negative for the presence of antibodies against SARS-CoV-2 collected and archived prior to 2015. The internal standard was developed using protocols provided by the Frederick National Laboratory courtesy of Ligia Pinto, PhD. Eight serum samples confirmed positive for the presence of antibodies against SARS-CoV-2 were pooled to create the internal standard. This was run on a SPIKE protein ELISA parallel to the Human SARS-CoV-2 Serology Standard provided by the Frederick National Laboratory. The assay was repeated two additional times on two different days and the results were sent to Dr. Troy Kemp at Frederick for analysis. The internal standard was determined to have a concentration of 2,615 binding antibody units (BAU) per mL. Separate day, inter-assay CVs for titers of IgG positive samples on three replicate plates was 13.9%. The average intra-assay CV for IgG titers was 6.5% (Supplemental Table 1). The percent coefficient of variability (%CV) of the antibody positive control was under 10% across plates and assay days.

The SARS-CoV-2 spike IgG assays were carried out in 384-well high-binding SpectraPlates (Perkin Elmer, Waltham, MA). Plates were coated with 13 µL full length spike protein (synthesized by Moffitt Cancer Center Chemical Biology Core Facility) at a 2 µg/mL concentration using a MultiFlo FX dispenser (Biotek, Winooski, VT) and were then sealed and stored at 4°C overnight. All plate washing (85 µL each cycle) and reagent additions were performed using the MultiFlo FX. The plates were next washed 3X with 1XPBST (0.1% Tween 20) and then blocked with 85 µL 1xPBST-3% nonfat dry milk for 1 hour. The plates were then washed once with 1xPBST-1% nonfat dry milk after which 40 µL of this buffer was added to all wells. Samples, controls and standards were then added in a 10 µL volume from the respective

© 2022 Giuliano AR et al. *JAMA Oncology.*

source plates to the assay plates using a Precision XS liquid handler (BioTek). The assay plates were then sealed and then incubated 2 hours at room temperature before being washed 3X with 1XPBST. Next, 13 µL of a 1:5,000 dilution of anti-human IgG detection Ab was added, sealed and incubated 1 hour at room temperature in the dark. The plates were then washed four times (1XPBST) before the addition of 25 µL OPD solution. After 15 minutes, the detection reaction was stopped with the addition of 13µL 5N H_2SO_4 and the well absorbance was read at a 492nm wavelength using a Neo 2 plate reader (BioTek). OD values for blank wells (no sample) were subtracted from all other OD values. Concentrations were determined using a linear regression of the standard OD values on a log-linear plot using Gen5 version 3.10 analysis software (BioTek).

Biostatistical methods

Seropositive rates were calculated for pre-vaccination, following receipt of the first and the second vaccine doses and their 95% exact confidence intervals were calculated based on the Binomial distribution. Seropositive rates were compared across patient characteristics using the Fisher exact test or Chi-square test as appropriate. To calculate the binding antibody IgG geometric mean titers (GMTs), the raw titer data were first log10 transformed, and then the mean and 95% confidence intervals of the log10-transformed data were calculated using the Student's t distribution, and finally the mean and 95% confidence intervals were transformed back to the original scale. The associations among SARS-CoV-2 antibody levels and patient characteristics were examined using Wilcoxon signed-rank test for comparison of two groups or Kruskal-Wallis test for comparison of multiple groups. Paired t-test was applied to examine the antibody level difference between two time points (e.g., Days 1 vs. 29, Days 29 vs. 57). All tests were 2-sided and the raw p-values were adjusted for multiple comparisons using the Bonferroni method. Adjusted p-values <0.05 were considered statistically significant. All the analyses were performed utilizing SAS (Version 9.4, SAS Institute Inc, Cary, NC) or R (Version 4.0.2).

© 2022 Giuliano AR et al. *JAMA Oncology.*

eTable 2. Percent Seropositive Before Vaccination and After Receipt of 1 and 2 Vaccine Doses

		Percent Positive (95% Confidence Interval)		
	**N**	**Day 1**	**Post-Dose 1**	**Post-Dose 2**
**Overall**	515	3.3 (1.9-5.2)	71.3 (67.1-75.1)	90.3 (87.4-92.7)
**Age Group (Median age 66 years)**				
<66	253	3.6 (1.6 - 6.6)	76.7 (71.0 - 81.7)	91.3 (87.1 - 94.5)
≥66	262	3.1 (1.3 - 5.9)	66.0 (59.9 - 71.7)	89.3 (84.9 - 92.8)
**Sex**				
Male	253	4.7 (2.5 - 8.1)	69.2 (63.1 - 74.8)	88.9 (84.4 - 92.5)
Female	262	1.9 (0.6 - 4.4)	73.3 (67.5 - 78.5)	91.6 (87.6 - 94.7)
**Race**				
White	479	3.1 (1.8 - 5.1)	70.8 (66.5 - 74.8)	89.8 (86.7 - 92.3)
Non-white	34	5.9 (0.7 - 19.7)	79.4 (62.1 - 91.3)	97.1 (84.7 - 99.9)
**Ethnicity**				
Hispanic	32	3.1 (0.1 - 16.2)	71.9 (53.3 - 86.3)	90.6 (75.0 – 98.0)
Non-Hispanic	482	3.3 (1.9 - 5.3)	71.4 (67.1 - 75.4)	90.2 (87.2 - 92.7)
**BMI**				
Normal (≤25)	153	3.3 (1.1 - 7.5)	73.2 (65.5 – 80.0)	92.8 (87.5 - 96.4)
Overweight (26-30)	170	2.4 (0.6 - 5.9)	71.2 (63.7 - 77.9)	89.4 (83.8 - 93.6)
Obese (>30)	190	4.2 (1.8 - 8.1)	69.5 (62.4 - 75.9)	88.9 (83.6 – 93.0)
**Primary Patient Category** [a]				
Hematological malignancies	301	3.7 (1.8 - 6.4)	60.1 (54.4 - 65.7)	84.7 (80.1 - 88.6)
Myeloid	93	2.2 (0.3 - 7.6)	65.6 (55.0 - 75.1)	92.5 (85.1 - 96.9)
AML	30	0.0	66.7 (47.2 - 82.7)	100.0 (88.4 - 100)
MDS, MPN, or MDS/MPN	49	2.0 (0.1 - 10.9)	61.2 (46.2 - 74.8)	85.7 (72.8 - 94.1)
CML	9	11.1 (0.3 - 48.2)	77.8 (40.0 - 97.2)	100.0 (66.4 - 100)
Other [b]	5	0.0	80.0 (28.4 - 99.5)	100.0 (47.8 - 100)
Lymphoid	110	2.7 (0.6 - 7.8)	49.1 (39.4 - 58.8)	70.0 (60.5 - 78.4)
ALL	7	0.0	42.9 (9.9 - 81.6)	85.7 (42.1 - 99.6)
CLL	23	0.0	21.7 (7.5 - 43.7)	65.2 (42.7 - 83.6)
Hodgkin Lymphoma	10	0.0	80.0 (44.4 - 97.5)	100.0 (69.2 - 100)
B-cell NHL	55	3.6 (0.4 - 12.5)	43.6 (30.3 - 57.7)	58.2 (44.1 - 71.3)
T-cell or NK-cell lymphoma	15	6.7 (0.2 - 31.9)	93.3 (68.1 - 99.8)	93.3 (68.1 - 99.8)
Plasma Cell	98	6.1 (2.3 - 12.9)	67.3 (57.1 - 76.5)	93.9 (87.1 - 97.7)
MM	88	6.8 (2.5 - 14.3)	65.9 (55.0 - 75.7)	93.2 (85.7 - 97.5)
Smoldering MM	6	0.0	83.3 (35.9 - 99.6)	100.0 (54.1 - 100)
MGUS	2	0.0	100.0 (15.8 - 100)	100.0 (15.8 - 100)

© 2022 Giuliano AR et al. *JAMA Oncology.*

Amyloidosis	2	0.0	50.0 (1.3 - 98.7)	100.0 (15.8 - 100)
Solid tumors	214	2.8 (1.0 – 6.0)	86.9 (81.6 - 91.1)	98.1 (95.3 - 99.5)
Breast	85	1.2 (0 - 6.4)	89.4 (80.8 – 95.0)	98.8 (93.6 - 100)
Cutaneous	25	4.0 (0.1 - 20.4)	96.0 (79.6 - 99.9)	96.0 (79.6 - 99.9)
Endocrine	11	0.0	72.7 (39.0 – 94.0)	100.0 (71.5 - 100)
Gastrointestinal	25	0.0	72.0 (50.6 - 87.9)	96.0 (79.6 - 99.9)
Genitourinary	45	4.4 (0.5 - 15.1)	93.3 (81.7 - 98.6)	100.0 (92.1 - 100)
Gynecological	1	0.0	0.0	100.0 (2.5 - 100)
Head and Neck	5	0.0	100.0 (47.8 - 100)	100.0 (47.8 - 100)
Sarcoma	5	20.0 (0.5 - 71.6)	60.0 (14.7 - 94.7)	100.0 (47.8 - 100)
Thoracic	11	9.1 (0.2 - 41.3)	81.8 (48.2 - 97.7)	90.9 (58.7 - 99.8)
Other	1	0.0	100.0 (2.5 - 100)	100.0 (2.5 - 100)
**Stage (for solid tumors only)**				
Early	103	1.9 (0.2 - 6.8)	92.2 (85.3 - 96.6)	99.0 (94.7 - 100)
Advanced [c]	110	3.6 (1.0 – 9.0)	81.8 (73.3 - 88.5)	97.3 (92.2 - 99.4)
**Disease status**				
Previously untreated	41	0.0	82.9 (67.9 - 92.8)	100.0 (91.4 - 100)
Remission	289	4.2 (2.2 - 7.1)	74.4 (69.0 - 79.3)	90.7 (86.7 - 93.8)
Relapse/refractory/stable disease	184	2.7 (0.9 - 6.2)	63.6 (56.2 - 70.5)	87.5 (81.8 - 91.9)
**Neutrophil count** [d]				
>1 x 10^9/L	441	3.6 (2.1 - 5.8)	68.0 (63.5 - 72.4)	88.9 (85.6 - 91.7)
≤1 x 10^9/L	11	0.0	100.0 (71.5 - 100)	100.0 (71.5 - 100)
**Lymphocyte count** [d]				
>1 x 10^9/L	311	3.9 (1.7 - 6.0)	75.9 (71.1 - 80.6)	92.6 (89.7 - 95.5)
≤1 x 10^9/L	141	2.8 (0.1 - 5.6)	53.2 (45.0 - 61.4)	81.6 (75.2 – 88.0)
**Among Plasma Cell Disorder and CLL patients (n=121)**				
**IgG level** [d]				
< 700 mg/dL	51	2.0 (0.0 - 10.4)	47.1 (32.9 - 61.5)	92.2 (81.1 - 97.8)
≥700 mg/dL	49	10.2 (3.4 - 22.2)	85.7 (72.8 - 94.1)	93.9 (83.1 - 98.7)
**IgA level** [d]				
<70 mg/dL	53	5.7 (1.2 - 15.7)	50.9 (36.8 - 64.9)	90.6 (79.3 - 96.9)
≥70 mg/dL	46	6.5 (1.4 - 17.9)	84.8 (71.1 - 93.7)	95.7 (85.2 - 99.5)
**IgM level** [d]				
< 40 mg/dL	75	6.7 (2.2 - 14.9)	58.7 (46.7 - 69.9)	90.7 (81.7 - 96.2)
≥40 mg/dL	24	4.2 (0.1 - 21.1)	91.7 (73.0 – 99.0)	100.0 (85.8 - 100)
**Received anticancer therapy within 3 months** [e]				
No	275	3.3 (1.5 - 6.1)	76.7 (71.3 - 81.6)	92.7 (89.0 - 95.5)
Yes	240	3.3 (1.4 - 6.5)	65.0 (58.6 – 71.0)	87.5 (82.6 - 91.4)

© 2022 Giuliano AR et al. *JAMA Oncology.*

**Chemotherapy** [f]	55	0.0	60.0 (45.9 – 73.0)	85.5 (73.3 - 93.5)
**Small molecules** [g]	119	5.0 (1.9 - 10.7)	64.7 (55.4 - 73.2)	87.4 (80.1 - 92.8)
Tyrosine kinase inhibitors (excluding BTK inhibitors)	28	7.1 (0.9 - 23.5)	78.6 (59.0 - 91.7)	92.9 (76.5 - 99.1)
BTK inhibitors	6	0.0	0.0	33.3 (4.3 - 77.7)
PI3K inhibitors	2	0.0	0.0	0.0
Proteasome inhibitors	24	0.0	50.0 (29.1 - 70.9)	100.0 (85.8 - 100)
Lenalidomide / pomalidomide	50	8.0 (2.2 - 19.2)	74.0 (59.7 - 85.4)	94.0 (83.5 - 98.7)
Venetoclax	6	0.0	16.7 (0.4 - 64.1)	50.0 (11.8 - 88.2))
Cyclosporine	1	0.0	100.0 (2.5 - 100)	100.0 (2.5 - 100)
**Anti-CD20 antibodies**				
Within 6 months	16	0.0	0.0	6.3 (0.2 - 30.2)
6-24 months	15	0.0	20.0 (4.3 - 48.1)	53.3 (26.6 - 78.7)
Not treated with Anti-CD20	484	3.5 (2.1-5.6)	75.2 (71.1-79.0)	94.2 (91.7-96.1)
**Anti-CD38 antibodies**				
Within 6 months	47	2.1 (0.1 - 11.3)	57.4 (42.2 - 71.7)	87.2 (74.3 - 95.2)
6-24 months	12	0.0	50.0 (21.1 - 78.9)	100.0 (73.5 - 100)
Not treated with Anti-CD38	456	3.5 (2.0-5.6)	73.2 (68.9 - 77.3)	90.4 (87.3 - 92.9)
**Radiation therapy**	18	16.7 (3.6 - 41.4)	72.2 (46.5 - 90.3)	88.9 (65.3 - 98.6)
**Immune Checkpoint inhibitors and cytokines**	23	0.0	69.6 (47.1 - 86.8)	100.0 (85.2 - 100)
**Patients treated with cellular therapy**				
Allo-HSCT any time prior to vaccination	63	1.6 (0.0 - 8.5)	57.1 (44.0 - 69.5)	92.1 (82.4 - 97.4)
Chronic graft versus host disease	49	2.0 (0.1 - 10.9)	49.0 (34.4 - 63.7)	89.8 (77.8 - 96.6)
On antirejection medications (tacrolimus, sirolimus, cyclosporine)	46	2.2 (0.1 - 11.5)	50.0 (34.9 - 65.1)	89.1 (76.4 - 96.4)
Auto-HSCT within the past 12 months	19	10.5 (1.3 - 33.1)	78.9 (54.4 - 93.9)	100.0 (82.4 - 100)
CD19-CAR-T any time prior to vaccination	8	0.0	12.5 (0.3 - 52.7)	12.5 (0.3 - 52.7)
BCMA-CAR-T any time prior to vaccination	4	0.0	75.0 (19.4 - 99.4)	100.0 (39.8 - 100)
**On prednisone >10mg/day for ≥10 days in the past 4 weeks**	48	2.1 (0.1 - 11.1)	39.6 (25.8 - 54.7)	87.5 (74.8 - 95.3)
**Line of systemic therapy to date**				
0	64	4.7 (0.4 - 10.8)	89.1 (78.8 - 95.5)	100.0 (94.4 - 100)
1	229	4.8 (2.1 - 7.9)	76.0 (69.9 - 81.4)	92.6 (88.4 - 95.6)
≥2	222	2.3 (0.7 - 5.2)	61.3 (54.5 - 67.7)	85.1 (79.8 - 89.5)

Blood draws were conducted on Day 1, prior to first vaccine dose; Day 29, prior to second vaccine dose; and Day 57, which was 4 weeks ±14 days following the second vaccine dose.

a. Fifty-nine patients had multiple cancers and were categorized according to the most active/severe cancer in the study investigators' opinion.

© 2022 Giuliano AR et al. *JAMA Oncology.*

b. Other includes AA, PNH, BPDCN, and histiocytic and dendritic cell neoplasms.

c. Solid tumors in advanced stage include stage 4 or locally advanced not usually considered curable by surgery or radiation therapy.

d. All labs were within 3 months before the first dose of vaccine. 12% of total patients were missing lymphocyte count. Among patients with plasma cell disorder or CLL,17% were missing IgG, 18% missing IgA, and 18% missing IgM.

e. Anti-androgen and anti-estrogen hormonal therapies were not included.

f. Chemotherapy includes conventional cytotoxic chemotherapy and hypomethylating agents.

g. Small molecules include tyrosine kinase inhibitors, proteasome inhibitors, lenalidomide, pomalidomide, and venetoclax.

Abbreviations:

Abbreviations: BMI, body mass index; AML, acute myeloid leukemia; MDS, myelodysplastic syndrome; MPN, Myeloproliferative Neoplasm; CML, chronic myeloid leukemia; AA, aplastic anemia; PNH, paroxysmal nocturnal hemoglobinuria; BPDCN, blastic plasmacytoid dendritic cell neoplasm; HL, Hodgkin Lymphoma; NHL, non-Hodgkin Lymphoma; ALL, acute lymphoblastic leukemia; CLL, chronic lymphocytic leukemia; MM, multiple myeloma; MGUS, monoclonal gammopathy of undetermined significance.

© 2022 Giuliano AR et al. *JAMA Oncology.*

eTable 3. Antibody Levels Before Vaccination and After Receipt of 1 and 2 Vaccine Doses

	**Geometric Mean (95% Confidence Interval)**		
	**Day 1**	**Post-Dose 1**	**Post-Dose 2**
**Overall**	13.7 (13.0 - 14.3)	98.2 (84.7 - 113.9)	1064.0 (902.3 - 1254.8)
**Age Group**			
<66	13.6 (12.8 - 14.5)	134.8 (109.2 - 166.4)	1334.6 (1061.7 - 1677.6)
≥66	13.7 (12.8 - 14.7)	72.4 (59.0 - 88.7)	854.9 (675.4 - 1082.2)
**Sex**			
Male	14.2 (13.1 - 15.4)	84.3 (68.3 - 104.2)	934.6 (732.4 - 1192.7)
Female	13.1 (12.5 - 13.8)	113.8 (92.4 - 140.1)	1206.0 (964.6 - 1507.7)
**Race**			
White	13.7 (13.0 - 14.3)	97.5 (83.6 - 113.7)	1025.5 (862.6 - 1219.1)
Non-white	13.8 (11.8 - 16.3)	110.1 (59.5 - 203.6)	1849.9 (1143.9 - 2991.4)
**Ethnicity**			
Hispanic	13.4 (11.6 - 15.6)	77.2 (43.3 - 137.7)	900.8 (491.6 - 1650.7)
Non-Hispanic	13.7 (13.0 - 14.4)	100.2 (85.9 - 116.9)	1083.7 (912.9 - 1286.3)
**BMI**			
Normal (≤25)	13.4 (12.4 - 14.3)	105.0 (80.1 - 137.8)	1165.0 (888.1 - 1528.2)
Overweight (26-30)	13.4 (12.4 - 14.5)	89.1 (69.2 - 114.7)	1006.1 (745.7 - 1357.3)
Obese (>30)	14.1 (12.9 - 15.5)	100.4 (77.9 - 129.3)	1038.3 (780.0 - 1382.2)
**Primary Patient Category** [a]			
Hematological malignancies	14.0 (13.0 - 15.0)	66.5 (54.4 - 81.3)	745.6 (579.0 - 960.2)
Myeloid	13.5 (11.9 - 15.2)	67.3 (47.2 - 95.8)	1285.8 (858.6 - 1925.7)
AML	12.5 (.)	55.3 (32.8 - 93.2)	1462.3 (824.3 - 2593.8)
MDS, MPN, or MDS/MPN	14.0 (11.2 - 17.5)	62.0 (37.0 - 104.0)	1022.2 (518.3 - 2015.9)
CML	14.9 (9.9 - 22.6)	153.7 (27.9 - 848.4)	1905.4 (940.5 - 3860.2)
Other [b]	12.5 (.)	109.4 (14.9 - 804.8)	2776.1 (957.9 - 8045.7)
Lymphoid	13.4 (12.2 - 14.7)	59.5 (41.5 - 85.2)	396.3 (242.9 - 646.7)
ALL	12.5 (.)	43.9 (10.0 - 192.3)	674.4 (61.4 - 7413.2)
CLL	12.5 (.)	21.1 (13.5 – 33.0)	208.5 (74.4 - 584.0)
Hodgkin Lymphoma	12.5 (.)	148.5 (38.1 - 578.7)	2513.1 (1291.5 - 4890.4)
B-cell NHL	14.2 (11.7 - 17.1)	60.1 (34.3 - 105.3)	240.6 (111.0 - 521.3)
T-cell or NK-cell lymphoma	13.3 (11.7 - 15.1)	175.2 (68.8 - 446.3)	1507.3 (661.5 - 3434.5)
Plasma Cell	15.0 (12.8 - 17.7)	74.5 (53.2 - 104.2)	903.6 (624.0 - 1308.6)
MM	15.4 (12.8 - 18.4)	71.7 (49.8 - 103.4)	898.3 (598.7 - 1347.9)
Smoldering MM	12.5 (.)	97.4 (39.9 - 237.7)	1108.8 (322.2 - 3816.3)
Solid tumors	13.3 (12.6 - 13.9)	170.1 (139.6 - 207.3)	1754.6 (1502.7 - 2048.8)

© 2022 Giuliano AR et al. *JAMA Oncology.*

Breast	12.8 (12.2 - 13.5)	212.3 (157.7 - 285.8)	2147.7 (1716.4 - 2687.4)
Cutaneous	13.0 (12.0 - 14.2)	224.5 (132.3 - 381.0)	1623.9 (934.1 - 2823.1)
Endocrine	12.5 (.)	136.3 (48.9 - 379.6)	1800.3 (945.6 - 3427.7)
Gastrointestinal	12.5 (.)	92.5 (49.5 - 172.7)	1033.7 (585.3 - 1825.7)
Genitourinary	14.0 (11.9-16.4)	157.4 (105.3 - 235.1)	1628.8 (1231.5 - 2154.3)
Gynecological	12.5 (.)	12.5 (.)	4303.5 (.)
Head and Neck	12.5 (.)	96.5 (22.2 - 419.0)	1603.4 (582.3 - 4415.2)
Sarcoma	16.7 (7.5 – 37.0)	45.3 (6.3 - 323.8)	987.9 (498.6 - 1957.6)
Thoracic	16.4 (8.9 - 30.1)	303.4 (70.0 - 1314.1)	2252.6 (622.2 - 8155.1)
Other	12.5 (.)	530.7 (.)	3632.3 (.)
**Stage (for solid tumors only)**			
Early	13.0 (12.2 - 13.8)	234.9 (181.8 - 303.5)	1965.2 (1595.6 - 2420.5)
Advanced [c]	13.5 (12.5 - 14.7)	123.8 (92.6 - 165.5)	1560.8 (1240.0 - 1964.6)
**Disease status**			
Previously untreated	12.5 (.)	168.2 (102.9 - 275.1)	2021.1 (1470.6 - 2777.7)
Remission	13.8 (12.9 - 14.7)	111.3 (91.5 - 135.3)	1265.0 (1009.4 - 1585.4)
Relapse/refractory/stable disease	13.8 (12.6 - 15.0)	71.0 (55.2 - 91.4)	701.0 (530.5 - 926.4)
**Neutrophil count** [d]			
>1 x 10^9/L	13.8 (13.1 - 14.6)	89.2 (75.7 - 105.1)	996.5 (827.9 - 1199.5)
≤1 x 10^9/L	12.5 (.)	107.5 (62.5 - 184.7)	1347.1 (347.4 - 5222.8)
**Lymphocyte count** [d]			
>1 x 10^9/L	14.0 (13.0 - 15.0)	117.4 (96.9 - 142.3)	1321.5 (1084.1 – 1611.0)
≤1 x 10^9/L	13.4 (12.4 - 14.5)	49.4 (37.6 - 64.7)	547.4 (375.7 - 797.7)
**Among Plasma Cell Disorder and CLL patients (n=121)**			
**IgG level** [d]			
< 700 mg/dL	13.4 (11.6 - 15.5)	35.0 (24.5 - 50.1)	494.7 (304.9 - 802.7)
≥700 mg/dL	16.8 (12.6 - 22.4)	148.3 (89.9 - 244.6)	1496.1 (892.1 - 2508.9)
**IgA level** [d]			
<70 mg/dL	15.3 (12.1 - 19.5)	37.8 (25.2 - 56.5)	591.7 (362.7 - 965.3)
≥70 mg/dL	14.7 (11.8 - 18.1)	152.6 (94.6 - 246.2)	1346.2 (782.7 - 2315.3)
**IgM level** [d]			
< 40 mg/dL	15.4 (12.6 - 18.9)	51.3 (35.3 - 74.4)	669.6 (431.8 - 1038.5)
≥40 mg/dL	13.8 (11.3 - 16.8)	211.2 (116.2 - 383.6)	1942.6 (1106.3 - 3411.3)
**Received anticancer therapy within 3 months** [e]			
No	13.6 (12.8 - 14.5)	116.9 (96.1 - 142.2)	1450.4 (1182.0 - 1779.6)
Yes	13.7 (12.8 - 14.7)	80.4 (64.3 - 100.7)	746.1 (575.6 - 967.1)
**Chemotherapy** [f]	12.5 (.)	70.8 (45.3 - 110.7)	628.5 (350.6 - 1126.5)

© 2022 Giuliano AR et al. *JAMA Oncology.*

**Small molecules [g]**	14.6 (12.8 - 16.7)	73.2 (53.4 - 100.2)	646.7 (441.9 - 946.5)
Tyrosine kinase inhibitors (excluding BTK inhibitors)	14.3 (11.8 - 17.4)	127.7 (63.3 - 257.6)	1444.1 (769.4 - 2710.2)
BTK inhibitors	12.5 (.)	12.5 (.)	27.7 (7.5 - 103.3)
PI3K inhibitors	12.5 (.)	12.5 (.)	12.5 (.)
Proteasome inhibitors	12.5 (.)	39.3 (22.8 - 67.6)	734.4 (364.0 - 1481.7)
Lenalidomide / pomalidomide	16.8 (12.5 - 22.7)	95.2 (56.9 - 159.3)	966.0 (561.9 - 1661.0)
Venetoclax	12.5 (.)	17.9 (7.1 - 44.8)	21.9 (7.0 - 68.4)
Cyclosporine	12.5 (.)	868.2 (.)	2807.4 (.)
**Anti-CD20 antibodies**			
Within 6 months	12.5 (.)	12.5 (.)	15.5 (9.8 - 24.5)
6-24 months	12.5 (.)	29.3 (12.1 - 71.0)	345.2 (60.1 - 1983.4)
Not treated with Anti-CD20	13.7 (13.1 - 14.4)	109.2 (93.9 - 126.9)	1267.1 (1088.7 - 1474.8)
**Anti-CD38 antibodies**			
Within 6 months	13.5 (11.6 - 15.7)	52.7 (33.2 - 83.4)	369.7 (219.7 - 622.1)
6-24 months	12.5 (.)	44.4 (17.4 - 113.2)	903.6 (292.3 - 2793.5)
Not treated with Anti-CD38	13.7 (13.0 - 14.4)	107.0 (91.3 - 125.3)	1191.6 (1001.3 - 1418.2)
**Radiation therapy**	19.6 (11.7 - 32.9)	138.5 (41.8 - 459.1)	1558.7 (564.7 - 4302.6)
**Immune Checkpoint inhibitors and cytokines**	12.5 (.)	77.1 (40.3 - 147.8)	1237.4 (815.3 - 1878.0)
**Patients treated with cellular therapy**			
Allo-HSCT any time prior to vaccination	13.6 (11.5 - 16.2)	47.7 (32.1 - 71.0)	1290.0 (762.0 - 2183.6)
Chronic graft versus host disease	14.0 (11.2 - 17.5)	38.4 (24.7 - 59.5)	1045.2 (552.0 - 1978.8)
On antirejection medications (tacrolimus, sirolimus, cyclosporine)	14.1 (11.1 - 17.9)	34.9 (23.6 - 51.7)	1099.3 (570.5 - 2118.0)
Auto-HSCT within the past 12 months	20.0 (10.1 - 39.5)	166.4 (60.7 - 456.1)	2452.6 (1080.1 - 5569.1)
CD19-CAR-T any time prior to vaccination	12.5 (.)	15.7 (9.1 - 27.1)	26.8 (4.4 - 162.9)
BCMA-CAR-T any time prior to vaccination	12.5 (.)	107.5 (9.5 - 1222.3)	2613.2 (1241.1 - 5502.2)
**On prednisone >10mg/day for ≥10 days in the past 4 weeks**	13.3 (11.7 - 15.1)	28.2 (18.6 - 42.8)	296.5 (167.6 - 524.4)
**Line of systemic therapy to date**			
0	13.3 (12.1 - 14.6)	204.5 (142.2 - 294.1)	2097.1 (1662.2 - 2645.9)
1	14.3 (13.0 - 15.7)	116.2 (92.9 - 145.3)	1315.5 (1041.1 - 1662.2)
≥2	13.1 (12.6 - 13.7)	66.9 (53.5 - 83.6)	703.0 (530.8 - 931.0)

a. Fifty-nine patients had multiple cancers and were categorized according to the most active/severe cancer in the study investigators' opinion.

b. Other includes AA, PNH, BPDCN, and histiocytic and dendritic cell neoplasms.

c. Solid tumors in advanced stage include stage 4 or locally advanced not usually considered curable by surgery or radiation therapy.

© 2022 Giuliano AR et al. *JAMA Oncology.*

d. All labs were within 3 months before the first dose of vaccine. 12% of total patients were missing lymphocyte count. Among patients with plasma cell disorder or CLL,17% were missing IgG, 18% missing IgA, and 18% missing IgM.

e. Anti-androgen and anti-estrogen hormonal therapies were not included.

f. Chemotherapy includes conventional cytotoxic chemotherapy and hypomethylating agents.

g. Small molecules include tyrosine kinase inhibitors, proteasome inhibitors, lenalidomide, pomalidomide, and venetoclax.

Abbreviations:

Abbreviations: BMI, body mass index; AML, acute myeloid leukemia; MDS, myelodysplastic syndrome; MPN, Myeloproliferative Neoplasm; CML, chronic myeloid leukemia; AA, aplastic anemia; PNH, paroxysmal nocturnal hemoglobinuria; BPDCN, blastic plasmacytoid dendritic cell neoplasm; HL, Hodgkin Lymphoma; NHL, non-Hodgkin Lymphoma; ALL, acute lymphoblastic leukemia; CLL, chronic lymphocytic leukemia; MM, multiple myeloma; MGUS, monoclonal gammopathy of undetermined significance

© 2022 Giuliano AR et al. *JAMA Oncology.*
